# Italian national consensus statement on management and pharmacological treatment of phenylketonuria

**DOI:** 10.1186/s13023-021-02086-8

**Published:** 2021-11-16

**Authors:** Alberto Burlina, Giacomo Biasucci, Maria Teresa Carbone, Chiara Cazzorla, Sabrina Paci, Francesca Pochiero, Marco Spada, Albina Tummolo, Juri Zuvadelli, Vincenzo Leuzzi

**Affiliations:** 1 Division of Inherited Metabolic Diseases, Reference Center for Expanded Newborn Screening, DIDAS Servizi Di Diagnostica Integrata, University Hospital Padova, 35128 Padua, Italy; 2 Maternal and Child Health Department, Pediatrics and Neonatology Unit, Guglielmo da Saliceto Hospital, 29121 Piacenza, Italy; 3 Pediatric Division, Metabolic and Rare Diseases, Santobono Pausilipon Hospital, 80122 Naples, Italy; 4 Paediatric Department, ASST Santi Paolo E Carlo, San Paolo Hospital, University of Milan, 20142 Milan, Italy; 5 Metabolic and Muscular Unit, A. Meyer Children’s Hospital, Florence, Italy; 6 Department of Pediatrics, Regina Margherita Children’s Hospital, University of Torino, 10126 Turin, Italy; 7 Metabolic Diseases Department, Clinical Genetics and Diabetology, Giovanni XXIII Children’s Hospital, 70126 Bari, Italy; 8 Department of Human Neuroscience, Unit of Child Neurology and Psychiatry, University La Sapienza, 00185 Rome, Italy

**Keywords:** Phenylketonuria, Italian consensus, Transition, Pharmacotherapy tailoring, BH4 test, Sapropterin, Pegvaliase

## Abstract

**Background:**

Phenylketonuria (PKU) is a rare inherited metabolic disorder caused by defects in the phenylalanine-hydroxylase gene (PAH), the enzyme catalyzing the conversion of phenylalanine to tyrosine. PAH impairment causes phenylalanine accumulation in the blood and brain, with a broad spectrum of pathophysiological and neurological consequences for patients. Prevalence of disease varies, with peaks in some regions and countries, including Italy. A recent expert survey described the real-life of clinical practice for PKU in Italy, revealing inhomogeneities in disease management, particularly concerning approach to pharmacotherapy with sapropterin hydrochloride, analogous of the natural PAH co-factor, allowing disease control in a subset of patients. Therefore, the purpose of this paper is to continue the work initiated with the expert survey paper, to provide national guidances aiming to harmonize and optimize patient care at a national level.

**Participants:**

The Consensus Group, convened by 10 Steering Committee members, consisted of a multidisciplinary crowd of 46 experts in the management of PKU in Italy.

**Consensus process:**

The Steering Committee met in a series of virtual meeting in order to discuss on clinical focuses to be developed and analyzed in guidance statements, on the basis of expert practice based evidence, large systematic literature review previously performed in the expert survey paper, and evidence based consensus published. Statements were re-discussed and refined during consensus conferences in the widest audience of experts, and finally submitted to the whole consensus group for a modified-Delphi voting.

**Results:**

Seventy three statements, divided in two main clinical areas, PKU management and Pharmacotherapy, achieved large consensus in a multidisciplinary group of expert in different aspects of disease. Importantly, these statements involve guidances for the use of sapropterin dihydrochloride, still not sufficiently implemented in Italy, and a set of good practice to approach the use of novel enzyme replacement treatment pegvaliase.

**Conclusions:**

This evidence-based consensus provides a minimum set of guidances for disease management to be implemented in all PKU centers. Moreover, these guidances represent the first statement for sapropterin dihydrochloride use, implementation and standardization in Italy, and a guide for approaching pegvaliase treatment at a national level on a consistent basis.

**Supplementary Information:**

The online version contains supplementary material available at 10.1186/s13023-021-02086-8.

## Background

Phenylketonuria is an autosomal recessive disorder caused by defects in the PAH gene, occurring in 1:10,000 live births in Europe, with higher prevalence in some countries, including Italy [1]. PAH catalyze the oxidation of phenylalanine (Phe) to tyrosine, thus, defects in PAH activity lead to impaired Phe metabolism and increase in blood concentration characterizing disease, with serious consequences for brain health [2]. The genetic landscape of PKU reveals more than 950 variants in PAH gene, causing different degrees of PAH loss of function and determining different clinical phenotypes, with blood Phe concentrations increasing with decreasing PAH residual activity [3]. On clinical ground, hyperphenylalaninemias (HPAs) are now classified according to the treatment options, including non-PKU HPA (Phe concentration ranging from 120 to 360 µmol/L), and PKU HPA (blood Phe concentration > 360 μmol/L) [4]. High blood Phe levels result in brain Phe accumulation which affects postnatal neurodevelopment, leading to severe and irreversible neurological and intellective impairment. Neonatal screening for PKU and early treatment have eradicated this clinical condition in countries with advanced health systems (https://www.osservatorioscreening.it/fenilchetonuria-screening-neonatale/). Current European guidelines recommend a lifelong treatment aimed at keeping blood Phe levels within the target range of 120–360 μmol/L until 12 years of age, to prevent neurodevelopmental derangement, and within 120–600 μmol/L later, in order to prevent possible neurocognitive decline or deterioration [5].

The milestone of PKU therapy is a strict low-Phe diet, requiring avoidance of high-protein foods, consumption of protein substitute distributed throughout the day and adequate energy intake, with significant patient burden [6]. Compliance is often poor, particularly during emotionally complicated adolescent years and in adults, when diet management also profoundly influences patient’s social relationships and quality of life [7].

In recent years, pharmacological treatments for PKU were developed, allowing to control blood Phe levels while easing or normalizing diet, opening the way to a possible alternative for PKU patients. Pharmacological treatment options include supplementation with sapropterin dihydrochloride, the synthetic form of tetrahydrobiopterin (BH4), the natural chaperone for PAH, and enzyme substitution therapy with phenylalanine-ammonia-lyase, pharmacologically active as the PEGylated form, pegvaliase.

Sapropterin dihydrochloride was the first pharmacological therapy developed for PKU, indicated in pediatric and adult patients with BH4 responsive-PKU [8], in association to diet therapy. BH4 activates residual PAH enzyme, improving oxidative metabolism of Phe, thus decreasing blood Phe levels and increasing Phe tolerance. Patients eligible for BH4 treatment can be identified based on a BH4 response test [9]. In responding patients with adequate metabolic control, sapropterin dihydrochloride treatment allows to significantly reduce blood Phe concentration while easing diet by increasing natural protein intake, leading to improved compliance and disease management [10]. Although sapropterin dihydrochloride was approved by the European Medicines Agency (EMA) in 2008 [11], clinical practice is still significantly variable among countries, particularly concerning BH4 response test, with a wide variety of protocols adopted in different countries and even within the same country [10, 12].

Pegvaliase, developed more recently, is based on a different mechanism of action, representing the first enzyme substitution therapy for PKU. Pegvaliase directly converts phenylalanine to ammonia and trans-cinnamic acid, regardless of PAH residual activity [13]. Pegvaliase treatment is indicated for adult PKU patients (> 16 years) with uncontrolled blood Phe levels above 600 μmol//L, on existing therapy [14]. In clinical trials, pegvaliase showed high clinical efficacy in reducing and maintaining blood Phe levels within target ranges, while allowing a diet with intact protein intake close to those recommended for general population. Improvement in inattention and mood symptoms were also observed with the use of pegvaliase [15]. Based on comparative clinical studies, pegvaliase was also defined by some authors as the most effective treatment option for adults with PKU who have difficulty keeping blood Phe ≤ 600 μmol/L with diet alone or diet + sapropterin [16]. Pegvaliase was approved for use in the EU in 2019 [17] and its use in clinical practice is currently being diffusing in most countries.

Thus, evidences show that enormous progresses in treatment of PKU patients were made in last years, although clinical practice largely differs among countries. The situation of PKU management in Eastern and Southern Europe was recently investigated in a survey study, outlining huge disparities in diagnosis and treatment in some countries and claiming for targeted efforts to optimize diagnostic and management approaches [18]. Accordingly, a recent survey study described the Italian real-life clinical experience of PKU management, with particular focus on management of adult PKU patients, Phe target ranges in different centers, therapeutic strategies, follow-up schedules and expectations regarding future treatments. The study revealed significant differences in PKU management among different specialistic centers in Italy, with particular issues concerning compliance to diet therapy in adult patients and heterogeneity in BH4 therapeutic approaches among different centers, highlighting the necessity of establishing a network of knowledge and experience among PKU specialistic centers, in order to optimize therapies and improve PKU patients life in Italy [10].

To this purpose, this article presents the first Italian Consensus on PKU management and pharmacotherapy, proposing alignment practices for ameliorating care throughout the country and approaching pegvaliase pharmacotherapy on a consistent basis, according to best evidences, expert opinion and current guidelines. In particular, the consensus program aimed to deeply investigate the conformity of current clinical practice in Italy with recent European guidelines, specifically addressing the opportunity to find a common approach among Italian centres and to apply best practice not yet implemented in real life. In this setting, guidance statements provided represent an integration between general guidances of European guidelines with peculiarities of clinical practice in Italy.

## Methods

### Development of guidance statements

This consensus process is the natural prosecution of an expert survey analysis on the state of art of PKU management and pharmacotherapy in Italy, culminated in the publication of an expert paper in 2021 [10], assessing relevant differences and gaps in PKU patients management among different specialistic centers, beside the lack of national guidances. A group of 10 health care practitioners from 8 specialistic centers in Italy, including pediatricians, metabolic specialists, neuropsychiatrists, psychologists and dieticians, with specific expertise in PKU care, was identified as the Steering Committee (SC) to guide the consensus process. All authors of the Italian expert paper were included in the SC to maintain continuity of the process. The SC met in a first Virtual Working Group Meeting where they identified a list of clinical topics to address and grouped the topics in two areas of clinical focus, PKU management and Pharmacotherapy. During the workshop, the SC shared experiences and knowledge on the topics, addressing peculiar experiences and clinical practice, in order to find an agreement on interventions needed to optimize clinical practice for PKU management and pharmacotherapy. Based on the discussion during the first meeting, guidance statements were developed by the SC and analyzed in a second virtual workshop, organized in two meetings, one per clinical focus. The SC then met in a series of virtual meetings to refine the guidance statements to be submitted for a modified-Delphi Consensus in a wider audience of experts.

The project was sponsored by BioMarin Pharmaceuticals. All SC meetings and consensus process were organized by CD Pharma, an independent communication agency, with no intervention of the sponsor in statements development or discussion.

### Consensus process

Guidance statements were analyzed in a first virtual consensus conference, divided in two sessions at a distance of one week, the first to discuss guidance statements related to PKU management and the second to analyze statements concerning pharmacotherapy. The SC shared the discussion with an audience of 36 experts from the main reference centers around Italy. In order to facilitate discussion, statements were divided in two groups in both conferences, and so were the SC and audience; each group of experts was assigned to a different working group/break-out room according to their particular expertise, where guidance statements were analyzed and refined to be submitted for consensus evaluation by the whole SC and audience. Working group A analyzed clinical topics related to Multidisciplinary Team, Nervous System Functioning, Neurocognitive and Psychiatric and Neuroimaging use in Follow-up, PKU Quality of Life; working group B, clinical topics concerning Predictive Value of the Genetic Profile, DBS Follow-up, Transition and Comorbidities, Adherence and Compliance; working group C discussed statements related to Sapropterin hydrochloride; working group D, statements related to Pharmacotherapy Tailoring and Pegvaliase.

Guidance statements were then sent to all participants for consensus evaluation in a modified Delphi survey, using a Survey Monkey platform. Participants expressed their level of agreement/disagreement on each statement anonymously, using a 5‐point Likert‐type scale (1 = strongly disagree, 2 = disagree, 3 = somewhat agree, 4 = agree and 5 = strongly agree). The number and percentage of participants who scored each item as 1 or 2 (disagreement), or as 3 (partial agreement) and as 4 or 5 (full agreement) were calculated. Consensus was considered to be reached when the sum for full agreement was ≥ 66%. All statements that achieved < 66% of full agreement in the first survey were further discussed by the SC during a virtual meeting. During this meeting, statements were refined and rephrased for clarification, to be proposed to all participants for further discussion in a second consensus conference. During the conference, statements were analyzed by all participants in a one part-session and further modified considering discussion and experts suggestions. According to the Delphi methodology, the statements were sent to all participants for a second round of consensus evaluation, using the same Survey Monkey platform and the same protocol for counting applied at the first round. Again, Consensus was considered to be obtained when the sum for full agreement was ≥ 66%.

## Results

A total of 73 guidance statements were developed after discussion among the SC about clinical topics related to management of patients with PKU and pharmacotherapy. After the first voting round, 59 statements reached consensus with ≥ 66% of full agreement. 14 statements scored from 39 to 65% of full agreement, thus, after further analysis in the SC, these statements were re-discussed, slightly changed and rephrased, to be submitted in a second consensus conference, obtaining full consensus.

Results presented below include the final guidance statements and a summary of the rationale behind guidances. A thorough dissertation about literature and discussion is provided for some clinical topics as supplementary material (Multidisciplinary team, Follow-up, Sapropterin dihydrochloride during pregnancy, Sapropterin efficacy, Pegvaliase induction, titration and maintenance strategy, Pegvaliase AEs prevention and management), leaving here arguments strictly related to statements development or resulting from particularly relevant discussion during consensus.

### PKU management

#### Multidisciplinary team

Current European guidelines provide the minimum requirements of the PKU multidisciplinary team established by the E.S.PKU [19]. Discussion among experts focused on the necessity of gathering all the indications of guidelines, in order to harmonize them with local practice and summarize them in guidance statements.

**Table:** 

**Statement # 1**
PKU needs to be managed through an integrated multidisciplinary transversal approach, all along patients’ life, throughout pediatric and adult care
**Statement # 2**
PKU multidisciplinary transversal team should include, as a minimum, one pediatrician with expertise in inherited metabolic diseases for care of infants and children, who should then accompany adolescents in the hands of an internist or metabolic expert for specific adult care, collaborating with at least one dietician and one psychologist
**Statement # 3**
Lab medical and genetics specialists, neuropsychiatrists and nurses are also needed to follow up clinical and pharmacotherapeutic management and outcomes
**Statement # 4**
Gynecological care is additionally required during childbearing age

#### Predictive value of the genetic profile

The degree of PKU severity is strictly related to the genetic abnormalities in PAH gene, affecting residual enzyme activity and determining a broad spectrum of phenotypes. Several allelic variants of PAH have been isolated and described, implementing databases (BIOPKU, http://www.biopku.org/home/biopku.asp) and genotype/phenotype association, thus providing a powerful tool for phenotype prediction, in order to implement tailored treatment strategies in PKU patients [20]. Recently, the statistical power of the BIOPKU database was investigated by using an allelic phenotype value (APV) algorithm to describe the relationship between genotype and phenotype in PKU. APV is a value defining the association of a variant with the corresponding metabolic phenotype, thus defining its severity, with APV = 0 corresponding to classic PKU, APV = 5 indicating mild PKU and APV = 10 mild HPA. Data revealed the high predictivity of APV and related genotypic-phenotype value, resulting in a genotype-based phenotype prediction of 99.2% for classic PKU, 46.2% for mild PKU and 89.5% for mild HPA [1, 21]. In Italy, patient genotyping is performed mostly to predict mild/moderate phenotypes for BH4 responsiveness [10]. As a matter of fact, genotype analysis should be considered in all PKU patients, in order to outline the metabolic phenotype and predict responsiveness to treatments [5].

**Table:** 

**Statement # 5**
PKU patient genotyping is required, as allelic variants can be useful to outline disease phenotype and the appropriate therapy tailoring
**Statement # 6**
International database on PKU might be helpful (BIOPKU database at http://www.biopku.org/home/biopku.asp) to understand PKU genetic profile analysis

### Follow-up

#### Dried blood spot (DBS) follow up

Dried blood spot (DBS) follow-up of patients with PKU is aimed to maintain Phe levels within the target range recommended, in order to prevent neuropsychological impairment. The consensus group agreed on the opportunity to align Italian practice to the recommendation of European guidelines [5], both concerning targeted ranges recommended and the schedule of DBS in different age ranges and conditions.

**Table:** 

**Statement # 7**	
Life-long systematic blood Phe level evaluation is recommended for PKU treated patients	
**Statement # 8**	
*Frequency of DBS follow-up for blood Phe testing should be adjusted according to age and patient’s condition:*	
0–1 year	Weekly
1–12 years	Every 2 weeks
> 12 years	Monthly
Women preconception	Weekly
Pregnancy	Twice weekly
**Statement # 9**	
In non-treated HPA patients DBS follow-up for blood Phe testing should be performed at least monthly, up to 2 years of age	
**Statement # 10**	
Frequency of DBS should be increased in particular conditions: treatment changes, intercurrent infections (gastroenteritis, fever), social or familiar changes (e.g. change of school/job, living home, family or work problems), pregnancy with specific requirements, adherence issues	
**Statement # 11**	
Target blood Phe ranges recommended are 120–360 µmol/L for 0–12 years patients and during pregnancy, 120–600 µmol/L after 12 years of age	

#### Nervous system functioning follow-up

PKU effects on postnatal development of the brain, as well as on mature brain functioning, are still not completely described [22]. High Phe levels (> 600 µmol/L) affect brain health in different ways, including both myelinization and neurotransmitters deficit (dopamine, serotonin), thus resulting in different effects on cognitive function and behavior, depending on the type of task analyzed and the age of patient [23], with peculiar neurocognitive deficits mostly associated to younger rather than to older patients [24]. For these reasons, the consensus’ group focused on the need of a tight schedule for nervous system functioning follow-up and the presence of neurology and psychiatry specialists and psychologists in the multidisciplinary team, as also recommended in current guidelines [5].

**Table:** 

**Statement # 12**
Phe levels affect nervous system health, with peculiarity related to specific patient physiology and overall effects on neurotransmitter depletion and increased brain vulnerability
**Statement # 13**
Patients with Phe values above defined safety range (120–600 µmol/L)—both early treated and late diagnosed—need to be tightly monitored, because of possible consequences on the nervous system, despite historical Phe
**Statement # 14**
The PKU multidisciplinary care team should include neuropsychiatrists, psychiatrists, psychologists and neurologists for routine evaluation of nervous system health and neurological damages in particular conditions

#### Neurocognitive and psychiatric follow-up

Neurocognitive studies in patients with early treated PKU confirm the relationship between metabolic control and the impairment in specific cognitive domines, as a result of Phe exposure in different developmental stages, throughout the life span [23]. In this framework, the necessity of implementing a shared schedule for neurocognitive and psychiatric follow-up of patients with PKU in Italy emerged, harmonizing any discrepancy in local practice with current guidelines [5] and best practices.

**Table:** 

**Statement # 15**
IQ full scale assessment is highly suggested at 12 and 18 years
**Statement # 16**
Neurocognitive and psychiatric follow up should be tailored according to patient’s clinical and life conditions, including but not limited to:
Worsening performance (school or work)
Comorbidities
Metabolic unbalance, due to dietetic or therapeutic incompliance
Psychiatric problems
Concerns of clinicians
**Statement # 17**
Neurocognitive assessment domains to evaluate according to patient’s clinical and life conditions are:
Planning, mental flexibility and problem solving
Inhibitory control (reaction time)
Visual-spatial memory
Visual-motor coordination
Short term memory /Working memory
Speed of processing
Sustained attention
Verbal fluency
Verbal memory and learning

#### Neuroimaging use in follow-up

Alterations on magnetic resonance imaging were observed both in early and late treated PKU patients [25]. Nonetheless, no long term evidences exist of a correlation between white matter or grey matter damage and Phe metabolic control. For this reason, waiting for further longitudinal studies, the consensus group agreed on the opportunity to follow the recommendation of European guidelines [5] and perform brain MRI only in patients with clear neurological alterations.

**Table:** 

**Statement # 18**
Brain MRI should be implemented in routine PKU care in case of clear neurological impairments

#### PKU quality of life

PKU strongly affects quality of life (QoL) of patients all along their life, also considerably impacting on their families and caregivers, bearing the responsibility of everyday care of children with PKU to prevent neurological damage [26, 27]. The strict diet therapy is a main daily struggle, with the stress related to preparation of food and obligation to a strict family organization representing an important burden for both patients and their families, importantly worsening their QoL [28]. Nevertheless, compliance to therapy is generally high during childhood, when diet is exclusively managed by parents, while diet management becomes particularly challenging during adolescence and later [6, 29]. Limits on socialization, perception of social isolation and the daily stigma imposed by diet are factors strongly affecting QoL of PKU patients, particularly because of problems related to self-esteem and the whole emotional sphere [30]. The requirement of lifelong follow-up may also represent further strains on patient’s QoL. A poor QoL generally leads to decreased confidence in treatments and, as a consequence, to poor compliance, with loss of follow-up and poor metabolic control, particularly in adolescents and adults [31]. Poor compliance thus increases the risk of cognitive and behavioral impairment, further intensifying the burden of illness (BOI) and, in turn, adding an additional load to patient’s QoL in a negative feed-back [32]. Based on these issues, experts agreed that QoL should be considered an all-encompassing aspect that needs to be investigated with a specific disease-related PKU-QOL instrument in PKU patients of all ages [10]. To this aim, the four PKU-QOL questionnaires, developed for different ages (Child PKU-QOL, Adolescent PKU-QOL, Adult PKU-QOL), and for parents of children with PKU (Parent PKU-QOL) represent valid and reliable instruments for assessing the multifaceted impact of PKU on patients of different age groups and their parents [26, 33].

**Table:** 

**Statement # 19**
QOL in PKU patients is an all-encompassing aspect, including the impact of clinical management and therapy adherence/compliance on the social and neurocognitive profile, following a virtuous circle modality, which needs to be investigated systematically with a PKU validated questionnaire—PKU-QOL specific for child, adolescent, adult or parents—addressing factors improving patient comfort, confidence in treatments, sociality and overall health
**Statement # 20**
PKU-QOL is dedicated to patients, families/caregivers, and tailored according to different needs for different age ranges—children, adolescents or adults
**Statement # 21**
PKU-QOL is highly suggested in chronic disease as part of clinical follow-up, and is useful to improve the overall management

#### Transition and comorbidities

Transition from childhood to adulthood is a difficult period for all adolescents, suffering emotional and social changes, in particular for young adults dealing with a metabolic disease like PKU and becoming more and more responsible of managing their condition. The main issue related to lack of follow-up during transition is patient perception of PKU as a pediatric disease and not as a lifelong condition [7]. Besides, in many countries, including Italy, adults with PKU continue to be cared in pediatric departments, a main cause of psychological and emotional discomfort for patients, leading to lack of confidence in the healthcare system, inconstant care and also loss of follow-up [7, 10, 18, 32]. Given these issues, any intervention to increase patient awareness about disease and the necessity of lifelong treatment, would be particularly beneficial for adolescents. To this aim all Italian centres agreed that PKU transition programs should be implemented toward an integrated, adult dedicated multidisciplinary team, to meet adult psychological requirements and clinical necessities, related to the overall BOI in adult patients with PKU that can lead to numerous comorbidities [34] and need to be specifically managed [10]. Basically, the discussion was leaded by experts from Italian centres that are approaching an implementation of the transition process, although with differences in protocol details. In particular, the necessity of working together on this topic was highlighted, in order to align to a common approach.

**Table:** 

**Statement # 22**
Transition from pediatric to adult care is a critical phase, due to changes in physiopathology, social life and overall care need
**Statement # 23**
Particular awareness and a structured care program are recommended during transition in order to match specific patient needs and expectations
**Statement # 24**
Adult specific care is also required in order to manage putative comorbidities emerging in adult patients—e.g. bone disease, diabetes risk, cardiovascular involvement

#### Adherence and compliance

PKU is mostly managed with the strict Phe-restricted diet therapy, considerably challenging and often difficult for patients to follow, particularly in adolescents and adults [6, 29]. Compliance is critically related to patient knowledge on his/her condition, and to factors influencing motivation and attitude [7, 32]. In this framework, it is crucial to implement a patient tailored approach to improve long-term adherence to diet treatment and prevent the nutritional, metabolic and cognitive abnormalities associated to non-compliance [6, 32, 35]. Accordingly, doctor-patient communication and relationship between patient and healthcare system should be seriously considered as a main factor facilitating adherence and engagement in the management of PKU, particularly during transition and in adults [5, 7, 10].

**Table:** 

**Statement # 25**
Adherence is a major concern in patients transitioning from childhood to adolescence to adulthood
**Statement # 26**
A patient tailored approach is recommended to achieve long-term compliance

### Pharmacotherapy

#### Pharmacotherapy tailoring

Pharmacotherapy provides an adjunct opportunity for patients with PKU to manage disease and maintain metabolic control. Both treatment with sapropterin dihydrochloride and pegvaliase allow to control Phe levels while relaxing or normalizing diet, with significative improvements in disease management and QoL [36].

Genotype–phenotype associations in PKU genetic databases provide a useful tool to predict the severity of phenotype [20] and responsiveness to sapropterin [3]. This is not the case for pegvaliase, since, as an enzyme substitution therapy, it is effective independently of the genetic profile [14]. Beside genotype–phenotype correlations, the main responsibility for clinicians is to make sure that pharmacotherapy is the most suitable treatment for a particular patient, both in term of effectiveness, feasibility and overall QoL. Moreover, it is important to considerate compliance and adherence to diet in those patients struggling with diet restrictions, resulting in poor metabolic control. Patients and their families/caregivers need to be aware about efficacy and potential benefits and risks, in order to best manage their expectations about treatment. Similarly, they have to be informed about how pharmacotherapy is administered and how therapy will influence their everyday life, in order to carefully consider their willingness of initiating and follow the treatment [37]. Definitely, particular attention is required when dealing with women in childbearing age and during pregnancy, carefully weighing possible benefits of pharmacotherapy in case of poor adherence to diet therapy [5].

Essentially, according to the group’s consensus, the choice of initiating pharmacotherapy requires a thorough evaluation of genotype and clinical/biochemical characteristics of the patient, the overall BOI, a comprehensive evaluation of patient’s attitude to change life habits and patient’s willingness of managing pharmacotherapy and testing schedule.

**Table:** 

**Statement # 27**
PKU pharmacotherapy should be initiated considering genetic profile, dietary compliance, gender, fertility, age of patients and family environment and support
**Statement # 28**
PKU pharmacotherapy should be considered in patients not able to be adherent, due the burdensome diet and overall burden of illness (BOI) that lead to persistent uncontrolled level of blood Phe
**Statement # 29**
PKU pharmacotherapy tailoring should consider patient clinical framework: BOI, patient willingness and life related feasibility
**Statement # 30**
Pharmacotherapy should be offered to patients considering the overall long-term compliance and adherence to prescribed treatment

#### Sapropterin dihydrochloride/BH4

Sapropterin was the first pharmacological treatment for PKU, approved by the Federal Drug Administration in 2007 and then by EMA in 2008 [11], providing an alternative to the strict low-Phe diet for patients with BH4 responding PKU. More than ten years of clinical practice provided reliable evidences for sapropterin efficacy and safety, so that guidances for patients testing and treatment were also included in last European guidelines [4, 5], Accordingly, sapropterin pharmacotherapy was discussed in consensus in order to align Italian practices to best recommendations and published guidelines.

#### Patient selection

Sapropterin is indicated in the treatment of HPA in patients of all ages who were shown to be responsive after a BH4 loading test [8]. Approximately 25% to 50% of patients with PKU respond to sapropterin [13].

The discussion focused on the necessity of considering overall patient’s characteristics beyond mere clinical parameters and genotype. An important issue to consider is patient’s (and family’s in case of children) real expectations and wills about therapy. For instance, they should be aware that responsive patients may still necessitate to follow a Phe-restricted diet together with sapropterin dihydrochloride, although diet will be less restrictive than without treatment [12, 37]. Also, in case of patients already on diet therapy, clinicians should accurately consider with them and their families the opportunity of modifying their consolidated dietary habits and lifestyle, to confirm their real willingness of initiating a different treatment [10].

Moreover, the use of sapropterin and criteria for testing largely differ across European countries and also within the same country [12]. In Italy as well, BH4 test is not routinely implemented for all patients in all PKU reference centers, with some center only testing patients with mild PKU and not all centers offering BH4 treatment to adult patients [10]. Thus, according to the group’s consensus, sapropterin treatment for Italian patients with PKU needs to be implemented, and criteria for treatment should be assimilated to those recommended in the European guidelines. Accordingly, BH4 treatment should be offered to all patients proven to be responders, for a most effective management of PKU compared to standard diet-therapy [5].

Concerning the use of sapropterin during pregnancy, cautions are recommended as for any pharmacological treatment [38]. Specific details of the discussion on this issue are provided as supplementary material.

**Table:** 

**Statement # 31**
BH_4_ treatment should be offered to all responding patients, due to a most effective disease management compared to standard dietary treatment
**Statement # 32**
BH4 treatment, allowing reduction in diet restrictions, should be offered to all responding patients, leading to an increase in long-term compliance and adherence to prescribed treatment
**Statement # 33**
Regarding the use of BH4 during pregnancy, specific awareness must be taken over the pregnancy trimesters about required blood Phe ranges
**Statement # 34**
BH4 treatment should be evaluated possibly before or at most during pregnancy, considering risks and benefits of initiating, interrupting or continuing treatment

#### BH4 genotype

According to current European guidelines, genotype is highly predictive of metabolic phenotype and BH4 responsiveness, thus all patients should be considered for BH4 responsiveness either by genotyping or with a BH4 response test [5]. Patients responding to BH4 generally present PAH allelic variants associated with mild-moderate phenotypes and only 1–2% of all HPAs are caused by pathogenic variants affecting the genes involved in BH4 metabolism [3]. It has been demonstrated that BH4 responsiveness is best predicted by APV values in 71% of patients analyzed in the BIOPKU database, providing a solid basis for suggesting genotype in all patients with a positive HPA diagnosis [12, 21]. BH4 responsiveness is associated to specific allelic variants, affecting the oligomerization of PAH multidomain arrangement that allows the complex regulation of the enzyme. Exogenous BH4 would partially or totally restore enzymatic activity, depending on allelic variants and degree of conformational impairment [39]. In patients carrying these mutations, BH4 responsiveness occurs almost in 80% of cases [40, 41]. Accordingly, in presence of a double known BH4 responding mutation, sapropterin treatment can be initiated without performing a BH4 response test [12]. After initial uncertainty of some experts about this issue, mainly due to discrepancies in clinical practice (agreement mostly equated disagreement after first voting), discussion during second consensus was particularly productive, with a thorough analysis of current literature and guidelines that finally led to a slight rephrasing of the statement (from “…test can be avoided…” to “…test may be avoided…”). The use of “may” was deemed more appropriate, since it leaves more space for individual decision, and the statement achieved full consensus. Importantly, BH4 responsive mutations were considered as predictive as PAH null mutations that correspond to non-responsiveness [41–43].

**Table:** 

**Statement # 35**
Genotype is highly suggested, as it can predict BH4 responsiveness with 71% accuracy
**Statement # 36**
Sapropterin should be used to treat PKU mild-moderate phenotypes, while classic phenotypes should be deeply investigated for allelic variants
**Statement # 37**
Patients with a double PAH null-mutations are not expected to respond to BH4; BH4 responsiveness test is not to be performed in these patients
**Statement # 38**
Patients with PKU sustained by a double mutation PAH, 100% responsive in BIOPKU, are BH4 responders; BH4 responsiveness test may be avoided in these patients and treatment started

#### Sapropterin efficacy

Approximately 25–50% of patients with PKU respond to sapropterin [13]. Most notably, sapropterin treatment allows to ease or normalize diet in some cases, by increasing Phe tolerance. This significantly helps patients and their caregivers in maintaining adherence to treatment, improving the everyday management of disease and QoL [2, 5, 10], as a main consequence of increased Phe control and improved neurocognitive outcomes [9, 44].

Sapropterin efficacy is assessed in a BH4 response trial by measuring blood Phe reduction in response to the administration of the drug. Efficacy is unanimously defined as a reduction of blood Phe concentration of at least 30% from baseline in a BH4 responsiveness test [5, 12, 45], as a lower threshold decrease (≥ 20%) was shown not to be predictive of true BH4 responsiveness [46]. In responding patients, sapropterin efficacy is also determined by an increase in Phe tolerance by a 100% rate [5, 12], allowing to introduce more natural protein in diet, while maintaining blood Phe levels within target ranges [47]. Discussion during consensus underlined the importance of considering both the decrease in blood Phe levels and the increase in Phe tolerance to identify one patient as a BH4 responder.

Importantly, sapropterin efficacy should always be evaluated on an individual case basis, considering overall benefits of treatment for the patient, beyond clinical improvements in Phe tolerance.

**Table:** 

**Statement # 39**
Sapropterin allows 30% in blood Phe reduction and 100% increase in Phe tolerance
**Statement # 40**
Therapeutic efficacy should be evaluated considering improvement in metabolic balance, neurocognitive profile and overall QoL
**Statement # 41**
Therapeutic efficacy should be evaluated considering less dietary restrictions and improvement in patient disease management, leading to better adherence and compliance

#### Pharmacokinetic

In Italy sapropterin is available as soluble tablets for oral administration [8]. Data show an improved bioavailability of sapropterin when administered with food, with a maximum blood concentration (maximum bioavailability) 4.5 h after drug administration with food [48]. Accordingly, DBS measures during short BH4 loading trials should be planned according to sapropterin pharmacokinetic, at least 4 h after drug administration.

**Table:** 

**Statement # 42**
During BH4 loading test for differential diagnosis between PAH and BH4 pathway deficiency, DBS testing should be started between 4 and 12 h after drug administration, according to sapropterin pharmacokinetic

### BH4 test

#### BH4 loading test

Historically, the BH4 loading test has been performed immediately after neonatal screening in order to distinguish patients with HPA due to PAH deficiency from patients with BH4 defects [45], although it is also used to identify BH4-responsive PKU, since BH4 treatment should be initiated as soon as possible after the HPA diagnosis [2]. There is no universally defined schedule for BH4 loading test and protocols widely vary among countries and even within the same country, from a 24–48 h test with administration of BH4 (10–20 mg/kg) every 24 h in Europe, to several weeks of administration with daily or weekly monitoring of blood Phe levels in the USA [38, 49]. Discussion during consensus compared opposite opinions, particularly on loading test purpose and timing, leading to borderline results for consensus during first voting round. The statements were rediscussed, referring to current guidelines and to assertions made in the Italian survey recently published, with a highly profitable debate that led to a wide acceptance. In Italy, BH4 test protocols slightly vary among different centers, although a 48 h schedule for BH4 loading is widely adopted for neonatal screening with 10 or 20 mg/kg of sapropterin [10]. According to the group’s consensus, a conventional 24 h protocol for loading test with 20 mg/kg of sapropterin should be used for differential diagnosis in neonates. Performing longer trials would delay initiation of treatment, possibly causing neurological damage in this delicate phase of neural development. As for blood Phe testing schedule, after basal DBS, 4, 8, 12 and 24 h samples were considered sufficient, according to conventional protocol of BH4 loading [50] and also considering that 16 h samples are difficult to collect in an out-patient setting.

**Table:** 

*Loading test patient selection*
**Statement # 43**
BH4 loading test should be performed as soon as possible after birth, following a positive HPA at newborn screening test
**Statement # 44**
BH4 loading test is used to primarily discriminate HPA sustained by BH4 deficiency from HPA caused by PAH loss of function
*Loading test execution*
**Statement # 45**
BH4 loading test should be performed in neonates or infants by a 20 mg/kg load monitored in 24 h, with blood Phe concentration measured at 0 h (pre), 4 h, 8 h, 12 h, 24 h. Breastfeeding or infant formula are maintained during the test

#### BH4 responsiveness test

Taking for granted all factors to ponder before considering BH4 pharmacotherapy, several protocols for BH4 test are adopted around the world and differences among Italian centers also emerged in the recent survey [10]. Discussion during consensus revealed the necessity to converge toward a widely shared protocol for BH4 testing, in order to optimize and standardize results, while offering the broadest opportunity for treatment to responding PKU patients. In neonatal patients the uppermost priority is initiating treatment with diet and/or sapropterin before day 10 [10, 12], thus a short BH4 test is most suitable. Conversely, non-neonatal patients already on diet therapy, who may not respond to BH4 in a shorter response test, a BH4 response test over 1 week or more, according to metabolic response, may be the preferred choice [12]. On this basis, any attempt to best fit BH4 test schedule with patient clinical characteristics and patient’s/parent’s feasibility and wills should be implemented, in order to optimize compliance, test reliability and benefits for the patient. After evaluation of clinical practice in PKU centers in Italy and comparison with current guidelines for BH4 test [12], experts widely agreed upon the opportunity of performing BH4 responsiveness test considering a short or a long protocol, for all patients, with specific cautions for pregnant women. Discussion also highlighted the importance of detecting Phe values before sapropterin administration [50] to evaluate the basal daily variation of blood Phe and compare it with BH4 induced variations during test. In case of no response in the 48 h or 1 week BH4 test, patients should be re-tested in a longer trial, up to 4 weeks, in order to detect late responders [10, 49, 51]. This particularly applies to patients with APV ≥ 5, since they have a high probability of response [12]. Duration of re-testing should not be mandatory but clinicians can decide on the basis of feasibility and response observed, by monitoring with DBS performed daily or twice a week. Following the proposal of some SC members, a dose range of 5–20 mg/kg/day was initially recommended in the guidance statement #52, based on the observation that some patients respond to a dosage as low as 5 mg/kg/day. Actually, this position was not widely accepted among participants and it was necessary to re-discuss statement #52 and restrict the recommended range for dosage adjustment between 10 and 20 mg/kg/day, according to Phe tolerance observed, as considered more indicated to observe a response to sapropterin, in alignment with most accepted evidences and recommendations [5, 49, 52], and prescribing information [8]. A further point of critical discussion was the timing of BH4 test for pregnant women. After initial hesitance of some experts about statement #53 and further discussion in second consensus conference, a wide agreement was achieved on the necessity of assessing BH4 responsiveness in those women who are unable to maintain Phe levels in target ranges with diet only. Responsiveness, if not tested before, should be assessed as soon as possible in a short test, in order to limit dangerous effects of high Phe levels for the fetus. According to the group’s consensus, a 48 h trial is the most suitable during pregnancy, both considering discussion on clinical practice and current guidelines [10, 12].

**Table:** 

*Responsiveness test patient selection*
**Statement # 46**
BH4 responsiveness test is aimed to evaluate the efficacy of exogenous BH4 co-factor to increase PAH residual enzymatic activity
**Statement # 47**
BH4 responsiveness test schedule should be decided according to patient clinical features and patient/family/caregiver willingness and feasibility
**Statement # 48**
BH4 responsiveness test should be considered in all patients not displaying a double null PAH mutation
**Statement # 49**
A previous negative BH4 loading or responsiveness test should not exclude re-testing, both in infants/children and adults once metabolism and diet are stabilized, in case of positive allelic phenotype values
*Responsiveness test execution*
**Statement # 50**
BH4 responsiveness test for neonates, infants, children, adolescents and non-pregnant adults can be performed in a short or in a long protocol:
The 48 h protocol with 20 mg/kg/day sapropterin dihydrochloride, with 2 sapropterin administrations at 0 and 24 h. Response is assessed by DBS Phe measurement at -24, -16, -12, the day before as baseline evaluation, then 0 h (pre), 4 h, 8 h, 12 h, 24 h after first sapropterin, and at 4 h, 8 h, 12 h, 24 h, after second administration;
The 1 week protocol with 10 or 20 mg/kg/day sapropterin dihydrochloride administration and DBS Phe concentration assessed daily, which can be prolonged up to 4 weeks, with Phe measurement from daily to bi-weekly
**Statement # 51**
No response in a 48 h test or in 1 week test does not exclude a late response, evaluated by a 4 week test, particularly in case of positive allelic phenotype values
**Statement # 52**
During BH4 response test, dosage can be titrated up or down between 10 and 20 mg/kg/day, to observe response
**Statement # 53**
A 48 h BH4 response test should be considered in pregnant women unable to maintain recommended blood Phe levels with a Phe-restricted diet
If patient is not responsive in the 48 h test, pharmacological treatment should be discontinued and patient managed with dietary treatment only
If patient responds to the 48 h test, sapropterin treatment is maintained adjusting the existing diet treatment

#### Dietary and drug maintenance and adjustment

Patients with PKU normally presents day by day oscillations in Phe tolerance, thus it is important that blood Phe values are stable before introducing adjustments in dietary Phe intake. For this reason, increase in dietary Phe should only be introduced following a systematic schedule, adding a fixed amount of Phe (i.e. 100 mg/day) per day and waiting at least 7 days to observe stable Phe tolerance before next adjustment [37]. In the SPARK clinical study by Muntau et al. [52], an algorithm for Phe intake adjustment according to mean Phe concentrations was proposed, advising adjustments every 2 weeks and introducing 5–15 mg of dietary Phe increase, according to patient’s phenotype and blood Phe concentration range observed. Also, it is important to consider that Phe tolerance is an individual parameter, strictly depending on PKU phenotype and growth. Current European guidelines recommend to increase Phe intake systematically by 50 mg/day, if blood phenylalanine levels are consistently maintained within the lower half of target blood Phe levels for at least 3 months (i.e. 120–240 μmol/L up to 12 years of age and 120–360 μmol/L if aged ≥ 12) [53]. A striking heterogeneity in clinical practice for diet adjustment in patients on sapropterin emerged during discussion among Italian experts. Diversity of protocols adopted in different centers was a main determinant of disagreement in the first phase of consensus, since statements were considered exceedingly strict and mandatory. On the basis of this diversity, the SC dietician specialist conducted an internal discussion among all dieticians participating in the process, and reformulated the statements proposing guidances aligned to best evidences and recommendations, while allowing clinicians and dieticians to fine-tune protocols for diet adjustment according to their consolidated clinical practice and to patient’s age and phenotype. Thus, titration schedules were finally formulated for 0–1 children or all older patients, and protocols were changed toward a wider flexibility to suit different clinical practices. As a result of re-discussion of protocols, all statements achieved full consensus after second voting round. Experts also agreed that diet titration should be performed without changing the composition and quality of (medical or natural) food, in order to minimize the psychological impact of diet change for patients, particularly in case new high-protein food is introduced and then a switch back to previous diet regimen is necessary [37]. Nevertheless, a gradual introduction of more natural protein in diet is desirable, since breastfeeding for infants with PKU is also encouraged in current European guidelines [5]. In older patients, introducing natural proteins in diet is associated with better acceptance, nutritional benefits, they are more efficiently utilized and, in turn, it translates in improvements in everyday management of diet, with psychological and social benefits. As for any diet adjustment, the amount of Phe introduced with natural food, protein substitutes or special food should be calculated according to standard tables and Phe tolerance tightly assessed by regular DBS measurement, waiting at least seven days before next diet change, to assess stable Phe tolerance [53].

**Table:** 

**Statement # 54**
During BH4 responsiveness test, diet should be initially maintained, keeping in mind basal Phe value, then natural protein intake should be titrated according to increase in Phe tolerance and overall efficacy
**Statement # 55**
Increase in natural protein intake should be performed among the same type and quality of foods already included in the diet, whenever possible
**Statement # 56**
Natural proteins requirement should be maximized and stabilized while protein substitutes gradually adapted accordingly
**Statement # 57**
In BH4 responsive patients, titration of dietary Phe intake should be done gradually, to assess maximum Phe tolerance
In children up to 1 year of age on BH4 therapy, if blood Phe levels are consistently maintained under the lower half of target Phe range (i.e. < 240 µmol/L), an increase of 5–10 mg/kg/day could be appropriate
For older children and adults, increasing Phe by 50–100 mg per day or up to a maximum of 20% of the current Phe intake should be considered
**Statement # 58**
For any dietary natural protein increase, it is highly suggested to wait minimum 7 days after last adjustment and enough related DBS results, before any further change

#### Pegvaliase

Polyethylene glycol (PEG)-Phenylalanine-Ammonia Lyase (PAL) or Pegvaliase is the first enzyme substitution therapy indicated to reduce blood Phe concentrations in adult patients (> 16 years) with blood Phe levels > 600 µm/L, who were unable to maintain recommended blood Phe levels with existing treatment [14].

Since the drug is still not available in Italy, a discussion on the best approach to pegvaliase treatment was considered appropriate in this work, analyzing prescribing information, best evidences and recommendation published.

#### Patient selection

Unless previously available pharmacological treatment for PKU, pegvaliase reduces blood phenylalanine concentrations by directly processing phenylalanine to trans-cinnamic acid [54], thus providing for enzymatic Phe processing in all PKU phenotypes, independently of PAH residual activity or BH4 chaperone. For this reason, pegvaliase is the only pharmacological treatment available for patients with severe classic PKU, determined by double PAH null-mutation [15]. According to these considerations, pegvaliase should be considered in all adult patients with PKU who have the ability to give informed consent to treatment and who the clinician considers able to adhere to treatment [55]. Importantly, discussion among experts also focused on factors to consider when deciding on pegvaliase treatment opportunity for a specific patient, beyond prescribing information. The main issue pointed out was the necessity of a comprehensive evaluation of clinical characteristics and an individual characterization of disease management of the patient before initiating treatment. Since pegvaliase is indicated for those patients who are not able to maintain Phe control with existing treatment [14], considering the ability of patient to manage with PKU and his/her problems in being adherent to previous treatment was considered of outstanding importance. Accordingly, it should be considered that pegvaliase would represent a therapeutical improvement for all those patients who are not able to attain to the Phe-restricted diet (alone or in combination to sapropterin), and clinicians should evaluate and discuss possible clinical and life-related benefits of pegvaliase treatment for the patient. In these patients the history of disease management and the associated BOI should be thoroughly evaluated, since pegvaliase could represent the opportunity to achieve metabolic control while improving significantly disease management and BOI. Clinicians should also consider particular problems with every day treatment management that could emerge in patients with neurocognitive problems [56]. In those patients, it is advisable to consider both possible problems in attaining to pegvaliase treatment and potential benefits of pegvaliase therapy for neuropsychological health observed in clinical studies [57]. Besides, since pegvaliase treatment has been associated with the risk of hypersensivity reactions, although not mandatory in prescribing information [14], the presence of a trained observer who can recognize and manage hypersensivity is highly suggested, as stated in current recommendations for pegvaliase use, based on evidence and consensus evaluation [55].

**Table:** 

**Statement # 59**
Pegvaliase is the only pharmacological approach available for classic PKU phenotypes sustained by two PAH-null mutations
**Statement # 60**
Pegvaliase treatment should be considered taking into account the difficulties in following diet regimen and be adherent to previous therapeutic approaches
**Statement # 61**
The overall BOI related to treatment history may represent an indication for the choice of initiating pegvaliase treatment
**Statement # 62**
Pegvaliase patient targeting should also evaluate neuropsychological health, life related feasibility and the presence of a trained care-giver

#### Pegvaliase therapeutical goals

Clinical efficacy and safety of pegvaliase treatment were thoroughly investigated in phase 1 to 3 clinical studies [16, 57–61]. Results showed that pegvaliase long-term administration was associated with sustained reductions in blood Phe concentration within recommended target range, while increasing natural protein intake to levels recommended for the general population, with an acceptable safety profile [15, 55, 57, 59, 61]. Clinical efficacy was also related with significant improvements in neurocognitive performance, with better mood and inattention scores, beside improved cognitive flexibility and working memory performance in patients on pegvaliase, related to lower blood Phe levels [15, 55, 57, 59]. Long term efficacy of pegvaliase was also demonstrated in extension studies, where patients treated with pegvaliase for 5 years obtained a sustained and stable reduction of blood Phe levels within recommended target range [60]. According to clinical studies, therapeutic response to pegvaliase should be defined when achieving a 20% reduction in blood Phe concentration from baseline, or a blood Phe concentration ≤ 600 μmol/L after 24 weeks [15, 16]. Clinical evidences also showed that time for response to pegvaliase treatment may vary in different patients, probably relating to patient’s immune condition [58]. Although most patients achieve therapeutical efficacy within 18 months of treatment, it could also take up to 30 months to achieve significant blood Phe reduction [14, 63]. Accordingly, discontinuation of therapy in patients not responding after 18 months could be considered. Besides, after discussion about prescribing information and recommendations, experts agreed that continuation of pegvaliase should be considered in those patients showing an overall benefit of treatment, beyond clinical values [14]. In fact, pegvaliase is effective in improving disease burden, due to beneficial effects on mood and inattention, and the enormous benefits of a natural diet, considerably ameliorating disease management and patient’s psycho-social condition, all factors to be seriously considered while evaluating pegvaliase efficacy [55].

Thus, after an extensive discussion on clinical studies and evidence based consensus guidances [15, 55], the consensus group agreed that pegvaliase treatment should be considered for life-long maintenance of physiological Phe values, also considering overall benefits of treatment for the patient and liberalization of diet.

**Table:** 

**Statement # 63**
Pegvaliase can lower Phe levels beyond the target range of < 600 µm/L, down to physiological values
**Statement # 64**
If a patient does not reach a clinically relevant blood phenylalanine reduction after 18 months of treatment, pegvaliase discontinuation should be considered
**Statement # 65**
The physician may decide, with the patient, to continue pegvaliase treatment in those patients who show beneficial effects (e.g. ability to increase protein intake from intact food or improvement of neurocognitive symptoms)
**Statement # 66**
Pegvaliase should be considered for life-long maintenance of blood Phe close to physiological while normalizing natural protein intake and liberalizing diet

#### Dietary and drug maintenance and adjustment

Pegvaliase is introduced following an induction, titration and maintenance schedule for dosage, under continuous supervision of a healthcare provider [14, 55]. Complying the SmCP [14], the induction-titration-maintenance schedule should be tailored according to patient’s response, reducing the dosage and increasing time schedule in case of tolerability concerns and adjusting dosage according to efficacy response.

At the same time, since pegvaliase substitutes PAH activity, allowing to reestablish normal Phe metabolism, pharmacological efficacy translates in normalization of Phe tolerance and diet can be liberalized consistently. According to prescribing information and current guidelines, patients should be followed by a clinician and a dietician for diet adjustment while on pegvaliase treatment. Consistently, diet should be initially maintained during induction, in order to observe improvement in Phe metabolism due to pegvaliase treatment [14, 55]. Main clinical studies indicate to advise patients to remain on existing diet during pegvaliase introduction and maintain dietary protein intake (from natural or medical food) within a 10% variation from baseline diet [57]. Blood Phe measurement during dose and diet titration should be performed every 1–4 weeks, and risk of hypophenylalaninemia should be carefully monitored, decreasing pegvaliase dosage or increasing dietary protein intake if necessary. In patients on a Phe-restricted diet, protein intake from natural food should gradually substitute protein from medical food until reaching the daily recommended intake for general population, by increasing intact protein by 10–20 g per step, according to metabolic response. In patients on unrestricted diet, pegvaliase dose titration is predominant and dosage should be reduced by 10–20% weekly, in case of persistent hypophenylalaninemia (blood Phe < 30 µmol/L in two consequent DBS) [55].

**Table:** 

**Statement # 67**
Patients should be counseled by dietitians to maintain consistent protein intake from natural food and medical food, within 10% of baseline, during induction and titration, to clearly evaluate pegvaliase impact on Phe metabolism
**Statement # 68**
Diet should also be adjusted during titration and maintenance, up to normalization
**Statement # 69**
Pegvaliase treatment should comply to Summary Characteristics of Product (SmCP) induction, titration and maintenance regimen and should be tailored, reducing dosage and increasing time schedule, according to patient’s tolerability, adverse reactions and efficacy response

#### Adverse events prevention and management

Pegvaliase determine immunogenic response in all patients, with the production of both anti-PEG and anti-PAL antibodies, both in the early (first 6 months) and late period of treatment (after 6 months) [58]. Anti-drug antibodies in circulation form drug-Ab complexes, whose presence is more significant during early pegvaliase treatment and then gradually decreases over time. Accordingly, initiation of treatment and titration are most critical for patient’s immune response, and particular attention should be dedicated to possible hypersensivity events in these phases [62]. Accordingly, pegvaliase is only available through a Risk Evaluation and Mitigation Strategies (REMS) program, in order to minimize and manage possible events of hypersensivity. The REMS also include strategies to minimize the risk of severe AEs. Clinical trials have shown that premedication is successful in preventing adverse hypersensivity reactions [57, 61, 62]. Premedication with an H1 antagonist, H2 antagonist, and antipyretic is required starting from the day before first administration and prior to each pegvaliase dose during induction and titration, while during maintenance, premedication should be considered on the basis of patient tolerability [14, 55, 62]. In case of acute severe hypersensivity reaction, pegvaliase interruption should be considered. Experts agreed that reintroduction of treatment could be evaluated by the clinician in case of less severe hypersensivity reaction, according to risk–benefit evaluation and considering referral to an allergy/immunology specialist. Also, rechallenge should be performed in a controlled setting to promptly intervene in case of acute reaction, and resuming premedication and trained observer should be evaluated by the clinician for the first weeks [14, 55]. Dose for rechallenge should be lower than before drop out and so should frequency of administration [55, 59].

**Table:** 

**Statement # 70**
Premedication is highly suggested to prevent hypersensitivity reactions among induction and titration of pegvaliase, with the administration of antihistamines (H1/H2 receptor antagonists, e.g. ranitidine, cetirizine, fexofenadine), with or without antipyretics or anti-inflammatory drugs (e.g. paracetamol, acetaminophen or ibuprofen) on a daily basis from the day before initiation
**Statement # 71**
Reduction or discontinuation of premedication may be considered, based on clinical judgment, once stable dosing is reached
**Statement # 72**
In case of severe hypersensitivity reactions during pegvaliase treatment, referral and/or supervision by an allergy/immunology specialist may be considered at the discretion of the prescribing metabolic specialist
**Statement # 73**
Rechallenge following acute systemic hypersensitivity reaction should be at a lower dose/frequency of pegvaliase than the last dose taken, considering resuming premedication and reinstate the trained caregiver, at the discretion of the prescribing metabolic specialist

## Discussion

The background of this consensus is in the survey analysis recently performed to describe the real life of PKU clinical management in Italy, revealing heterogeneity of clinical practice among different centers, particularly concerning PKU pharmacotherapy [10]. Also, as for all rare diseases, the challenge of small data is a further obstacle for clinicians on the road to standard clinical practice. Therefore, with this process we aim to fill the gap of poor and inconsistent data on clinical practice at a national level and the lack of clinical guidelines for PKU care in Italy. Particularly, the consensus aims to provide standard guidances to optimize management of PKU patients at a national level and widen knowledge on pharmacotherapies now available for treating PKU patients. In this framework, the consensus was a main opportunity for comparing different positions coming from main PKU centers in Italy, more aligned on updated clinical guidelines, and smaller reference hospitals, sometimes attached to old positions and protocols, particularly concerning sapropterin treatment. Some statements didn’t achieve consensus after first voting round and required a thorough analysis, re-discussion during second conference, and some of them were also reformulated for clarifying concepts and/or incorporate different practices, before second voting. This process represented an extremely valuable occasion of detailed study and discussion on most recent guidelines, evidences and observations emerged from the last Italian survey [10]. As a result, the consensus had a highly formative role, providing a widest audience with the opportunity of reconsidering dated positions and evaluate best practice and guidelines in the context of real-life clinical practice.

All guidance statements were developed, phrased and fine-tuned exclusively by the SC on the basis of discussion about expert practice based evidence, large systematic literature review and evidence based consensus published, without any influence from the sponsor. Moreover, the whole consensus process was managed by an independent communication agency, in order to maintain editorial independence from the sponsor. Experts were enrolled on the basis of their particular expertise in PKU management. Particularly, given the complexity of PKU, healthcare professionals with diverse medical education were invited and, considering heterogeneity of clinical practice in different reference hospitals, experts from different centers all around the country were enrolled in the process. Our conference process methodology provides reliability and strength to the consensus due to the diverse medical background and geographic localization of the SC members and expert audience, beside the anonymous voting method, conferring affordability to the crowd of experts and ensuring that voting decisions were taken independently, without biases, in accordance with the Delphi methodology [63]. Further validating the methodology for consensus, all statements proposed by the SC reached a wide consensus after first round of voting or after re-discussion and second voting, also confirming adequacy of clinical topics discussed. Notably, a main purpose of the consensus process was the discussion of caveats related to lack in standard practice in some healthcare institutions. In fact, experts participating to consensus came from medical institutions of various typologies, from district hospitals to University institutions with research facilities and availability of multidisciplinary competences collaborating in PKU care. This allowed to identify and discuss in consensus conferences points of strength and weakness in PKU management in Italy and specifically address meaningful guidances that need to be implemented in all centers, as considered minimum essential requirements for optimal PKU care.

In particular, the necessity of aligning to most recent European guidelines for general management of patients with PKU definitely emerged from discussion and was confirmed by statements agreement. The urgency to converge toward standard requirements of a multidisciplinary team encompassing all aspects of disease, including transversal follow up of patients from childhood to adulthood with systematic implementation of a structured transition, was a mainstay of discussion about PKU management. Although some experts claimed difficulties related to the composition of the team because of lack of adequate healthcare resources and sufficient facilities for complete multidisciplinary care, the need of overcoming these limitations was definitely agreed in consensus, also involving executive and administrative figures to implement healthcare staffs, where necessary. Similar caveats were analyzed during discussion on follow up guidances, particularly concerning nervous system functioning follow up, underlying the requirement for standardization of neurocognitive areas to evaluate. Essentially, if clinicians decide which neurocognitive test to use, according to clinical experience or facilities available in the center, the consensus’ group declared that a comprehensive neuropsychological evaluation exploring cognitive performance across different domains is essential for an optimum follow up of patients with PKU. Providing guidance on critical neuropsychological domains to evaluate, this consensus would also consent centers to collect a minimum set of meaningful information about standardized areas of investigation.

Discussion about pharmacotherapy for PKU patients focused on critical aspects to consider when deciding on pharmacological treatment. Although statements developed from this consensus are not intended as clinical guidelines, the consensus’ group aims to provide ‘best guidances’ for an effective implementation of pharmacotherapy for PKU at a national level. As a matter of fact, PKU has been managed exclusively with diet-treatment for decades and some patients clearly manifest diffidence when they are proposed to change disease management. Also, a certain prudence may also be perceived in some clinicians, due to loyalty in consolidated clinical practice. Accordingly, clinical practice related to sapropterin use in Italy is no doubt to be implemented and standardized. This consensus provides for the first time a set of guidances for sapropterin use in responder patients with PKU in Italy, focusing on the importance of a patient tailored approach, also considering implementation of patient’s genotyping, generally not standardized so far. The consensus’ group definitely claimed that, considering mounting evidence on benefits of sapropterin treatment in responding patients, most efforts are needed to overcome any barrier to full implementation of sapropterin use and align to current guidances, in order to offer to all eligible patients the best therapeutical option to improve their health and overall wellbeing.

A last part of consensus evaluation was dedicated to the introduction of pegvaliase treatment in clinical practice in Italy. The aim of guidance statements on pegvaliase treatment for adult patients with PKU is to provide professionals involved in PKU care with a set of ‘wise practice’ for approaching this novel therapy at a national level. Since pegvaliase was not available in Italy at the time of consensus, none of the members of the SC or audience had direct clinical practice experience of pegvaliase use. Thus, this consensus was the opportunity for a peer to peer discussion to agree on the best approach to this novel pharmacotherapy at a national level. In particular, the discussion focused on more variable aspects of the therapy related to the diversity of the disease. Guidances are based on the analysis of systematic literature review and evidence- and consensus-based recommendations for pegvaliase use [55]. Moreover, SC experts tightly referred to pegvaliase prescribing information during development of guidances, including the management of adverse events. The SC agreed that an important part of decision about treatment should be left to professional judgement, particularly concerning initiation of treatment and rechallenge in case of discontinuation, mainly considering the whole disease history of the patient, possible benefits deriving from treatment and diet liberalization, and the risk/benefit ratio. It is also important to consider that reference literature and clinical studies on pegvaliase efficacy and safety cited in this consensus pertain exclusively to pegvaliase experience in the USA; translating this experience in other countries, including Italy, will be necessary to understand the real impact of pegvaliase treatment on management of PKU patients in specific clinical practice and real life.

## Conclusions

This evidence-based consensus aims to fill the gap of inconsistent clinical practice for patients with PKU in Italy, providing a minimum set of guidances for disease management, considered essential requirements for optimal patient’s care. The statements cover wide aspects of PKU care, primarily related to target Phe levels for treatment, treatment tailoring, treatment goals and follow-up.

Most importantly, this is the first consensus on the use of sapropterin in patients with BH4 responding PKU in Italy, providing a basis for implementation and standardization of this treatment at a national level. The consensus also provides a set of wide practices to approach the use of pegvaliase in adult patients, in order to introduce the treatment in clinical practice in Italy on a consistent basis.

The guidances are intended for clinicians, lab medical/genetics specialists and paramedical specialists involved in the care of patients with PKU, to help to harmonize clinical practice and to standardize and improve care.

## Supplementary Information

**Additional file 1:** A thorough dissertation about literature and discussion among the SC for some clinical topics: Multidisciplinary team, Follow-up, Sapropterin dihydrochloride during pregnancy, Sapropterin efficacy, Pegvaliase induction, titration and maintenance strategy, Pegvaliase AEs prevention and management.

## Data Availability

Data sharing not applicable to this article as no datasets were produced or analyzed during the study.

## Abbreviations

AEs: Adverse events
APV: Allelic phenotype value
BH4: Tetrahydrobiopterin
BOI: Burden of illness
DBS: Dried blood spot
E.S.PKU: European Society for Phenylketonuria and Allied Disorders
EMA: European Medicines Agency
HPA: Hyperphenylalaninemia
IQ: Intelligence quotient
MPKU: Maternal PKU
MRI: Magnetic resonance imaging
PAH: Phenylalanine-hydroxylase
PAL: Phenylalanine-ammonia lyase
PEG: Polyethylene glycol
Phe: Phenylalanine
PKU: Phenylketonuria
QoL: Quality of life
REMS: Risk evaluation and mitigation strategies
SC: Steering Committee
