# Supplementary material for: Italian national consensus statement on management and pharmacological treatment of phenylketonuria
Source: Orphanet J Rare Dis. 2021 Nov 16;16:476. doi: 10.1186/s13023-021-02086-8 (PMC8594187; doi:10.1186/s13023-021-02086-8)
Supplement: Supplementary file 1 — Additional file 1: A thorough dissertation about literature and discussion among the SC for some clinical topics: Multidisciplinary team, Follow-up, Sapropterin dihydrochloride during pregnancy, Sapropterin efficacy, Pegvaliase induction, titration and maintenance strategy, Pegvaliase AEs prevention and management. [file 13023_2021_2086_MOESM1_ESM.docx]

# Supplementary material

### Multidisciplinary team

PKU is a life-long metabolic disorder causing various degrees of impaired Phe metabolism. The significant effects of Phe levels on several neurocognitive scores support the role of metabolic control in affecting nervous system development and cognition, and underline the need of a continuous control of Phe metabolism both in children and adult patients with PKU [1]. Accordingly, current European guidelines confirm minimum requirements of the PKU multidisciplinary team established by the E.S.PKU [2], and highlight the need of implementing a structured transition process toward an adult care team [3]. At a national level, the real life of PKU management in Italy has been recently depicted in the survey of six main specialistic centers for PKU patients [4], which have an internal team composed by at least one medical specialist, one pediatrician or neuro-pediatrician, almost always supported by a dietician and a psychologist. The survey also revealed lack of structured transition owing to patient’s discomfort and discontinuation in follow-up, beside the lack of specific adult care [4, 5,]. Accordingly, the consensus group underlined the importance of a gradual switch of patients during transition, in order to improve patient’s comfort and confidence in care and manage specific aspects of disease and comorbidities related to the adult age. Also, the presence of a gynecological specialist integrated in the team is desirable for PKU patients during childbearing-age and particularly during pregnancy, to focus on risks connected with pregnancy in women with PKU and poor metabolic control, and follow specific patient requirements through different stages of pregnancy [4].

### Follow-up

**Dried blood spot (DBS) follow up**

The primary goal of follow up for PKU patients is to avoid neurocognitive impairment due to high blood Phe levels. The relationship between blood Phe levels and neurocognitive outcomes is largely described in literature, guiding the choice of standard blood Phe concentration recommended for a normal neurocognitive development, at any life stage [3]. Particularly, a stronger association is observed between blood Phe levels >320 µmol/L at early life stages (0-13 years) and the risk of having an IQ<85 in later years, as shown in meta-analysis studies [6, 7]. Taken together, evidences strongly indicate that for patients <12 years of age a Phe concentration of 360 μmol/L should be considered as the upper target blood Phe concentration to avoid neuropsychological detriment [3]. Similarly, Albrecht et al. [6] illustrate no differences in neuropsychological speed tests of 7 different categories between adolescents with PKU aged 13-18 and healthy controls, when blood Phe concentration are maintained below 570 μmol/L. In accordance, a range of 120-600 µmol/L is the target recommended in the European guidelines for PKU patients >12 years [3]. Several methods exist to assess blood Phe concentration, with negligible differences, and currently DBS is widely the most used [8]. Concerning the frequency of DBS, discussion during consensus underlined significant differences among different Italian centers, thus experts initially decided to state a schedule that could integrate all these heterogeneities, recommending a tight DBS evaluation until 2 years of age (once per week), possibly relaxing it to 1-2 weekly in 3-6 years old children, then passing to fortnightly until 12 years of age, and to monthly after 12 years of age. Several experts disagreed with this tight DBS schedule, considered overstressing, particularly for infant patients. Moreover, children of this age are under the rigorous control of parents, with adherence to therapy and metabolic control generally adequate, thus unnecessary distressing follow up should be avoided. Reflecting these differences in opinion, this statement didn’t achieve consensus after first voting and was reformulated for the second Delphi round toward an alignment with current European guidelines, recommending a tight DBS monitoring (once per week) only for children aged 0-1 years. Once treatment and follow up are established, frequency can be decreased to fortnightly, between 1 and 12 years of age, and monthly for patients older than 12 years [3]. In accordance to guidelines, same target Phe ranges and frequency of DBS follow up were described in the Italian survey study by Burlina et al. [4].

Particular conditions, such as pregnant women with PKU, require specific attention, since high blood Phe levels during pregnancy may cause maternal PKU (MPKU) syndrome, with possible teratogenic effect on the fetus, leading to growth retardation, microcephaly, intellectual disabilities and birth defects [9]. At this regard, the MPKU international study displayed good child outcomes in mothers with normal IQ and blood Phe concentration constantly maintained between 120 and 360 μmol/L during all pregnancy [10]. Accordingly, the same Phe target range is recommended in European guidelines [3]. A particular accuracy in MPKU follow-up was also found in all centers analyzed in the recent Italian survey [4].

Blood Phe concentration should be routinely tested also in patients with HPA not requiring treatment, in order to check for maintenance of Phe tolerance with growth [11]. To this aim, performing DBS testing in these patients at least monthly up to 2 years of age seems appropriated.

**Neurocognitive and psychiatric follow-up**

Despite a large interindividual variability of clinical outcome in adulthood, neurocognitive studies in patients with early treated PKU confirm the relationship between metabolic control and the impairment in specific cognitive domines, as a result of Phe exposure in different developmental stages, throughout the life span [12]. Maintaining Phe levels in recommended ranges with treatment is expected to be able to prevent this kind of late neurocognitive impairment. According to European guidelines, neurocognitive routine evaluation should be performed at 12 and 18 years, corresponding to changes in treatment target for blood Phe and/or life changes (school, living situation, job, transition to adult care). Accordingly, neurocognitive assessments should also be tailored according to patient’s condition and complaints, emerging neurological and/or psychiatric symptoms, or decline of performances in her/his everyday life. To this aim, the presence of neuropsychiatrists, psychiatrists, psychologists and neurologists integrated in the PKU multidisciplinary team should be recommended, even if the matter is still challenging in most country [3, 4].

**Neuroimaging use in follow-up**

White matter (WM) alterations on magnetic resonance imaging (MRI) were described both in early and late treated PKU patients [13]. WM involvement is negatively related to adherence to diet and metabolic control, and is generally more evident in older patients [14, 15]. Although the linkage between WM alterations, as detected by conventional MRI, and cognitive outcomes is inconsistent [16, 17], WM abnormalities and cognitive deficits can be reversed by lowering blood Phe concentrations [18, 19]. In adults with PKU, elevated blood Phe levels have been associated to biogenic amine neurotransmitter reduction in the cerebrospinal fluid, and to specific grey matter atrophy patterns at MRI [20]. Further studies provide evidence for a possible correlation between Phe levels and clinical, neuropsychological, neurophysiological, biochemical, and neuroimaging alterations in early treated PKU patients during adulthood [16, 21]. A further implementation of MRI in longitudinal studies would be necessary for a deeper understanding of brain damage patterns in relation to Phe metabolic control. Waiting for results of longitudinal studies, at present neuroimaging techniques are not considered useful in routine care of patients with PKU, thus brain MRI should be reserved for those patients presenting with an atypical clinical course and/or unexpected neurological deficits [3].

### Sapropterin dihydrochloride during pregnancy

A certain reluctance to use sapropterin during pregnancy emerged from debate among experts. Several hesitations arose after first voting round (borderline result for statement #34) and the consensus represented a viaticum to widen knowledge on risks and benefits of sapropterin in pregnant women. Analysis of data from the PKU-MOM sub-registry, showing that sapropterin is effective in reducing blood Phe levels in pregnant women and is normally well tolerated [22], beside discussion on recent guidelines, strongly recommending dietary treatment and/or BH4 therapy as essential both pre-conceptionally and during pregnancy [3, 23], were decisive to align the audience on the opportunity of sapropterin treatment during pregnancy, if metabolic control is not maintained with dietary treatment. Also, the central role of preventive measures (pregnancy planning, preconception counselling) was reaffirmed. As a matter of fact, if preconception counselling and follow-up are implemented, it is quite infrequent for clinicians to evaluate BH4 treatment for women during pregnancy. On these bases, statement #34 was left unchanged and further discussed to clarify hesitations. After second voting round, experts widely agreed that BH4 responsiveness should be assessed preferably preconceptionally, and that it can be done during pregnancy only in case of scarce metabolic control. The same applies in case of women already on sapropterin treatment, for whom clinicians will evaluate risks and benefits of continuing or interrupting treatment, according to metabolic control. Also, particular attention should be given to the trimester of pregnancy. Indeed, maternal blood Phe concentrations exhibit different effects according to phases of fetus development, thus maternal Phe levels have an influence throughout all pregnancy [9], and a strict metabolic control is fundamental for positive birth outcomes [24].

### Sapropterin efficacy

Due to increased metabolic control, sapropterin allows to unburden diet, decrease the use of medical food, thus improving compliance, disease management and QoL [25, 26], as also demonstrated in longitudinal studies [27]. Also, although at present long-term neurocognitive outcomes in response to sapropterin have not been reported, several indications exist of a positive effect of sapropterin treatment on nervous system development and functioning, in term of improvement in WM integrity, executive abilities [19] and preservation of normal IQ scores [28]. Based on these issues, sapropterin efficacy should be evaluated in a comprehensive context of patient management, also considering neuropsychological improvements deriving from treatment.

### Pegvaliase dietary and drug maintenance and adjustment

According to prescribing information, pegvaliase is introduced following an induction, titration and maintenance schedule for dosage, under continuous supervision of a healthcare provider [29, 30]. Finding the right dosage to obtain significant results might take several months in some individuals. Although most patients achieve therapeutical efficacy within 18 months of pegvaliase treatment, it could also take up to 30 months to achieve significant blood Phe reduction [29, 31]. As for prescribing information, the induction dosage for pegvaliase should be 2.5 mg subcutaneously once per week for 4 weeks, in order to induce immune-tolerability [29, 32]. After induction, titration to target maintenance dosage should be performed step by step. Prescribing information recommend titration of dosage to 20 mg/day in a minimum of 9 weeks, although patient’s individual response to drug and tolerability should be considered. Actually, some individual may have a more pronounced immune-response to pegvaliase and the clinician should accurately evaluate tolerance in this subjects. [29, 30]. Published data suggest that change in immune response over time influences the dosing necessary to achieve efficacy [33]. Consistently, while some patients show an efficient blood Phe concentration reduction at the lowest dosage during titration and do not need any further increase, others may show a delayed response and need more time before increasing pegvaliase dosage [29, 30]. In the PRISM-1 clinical study, 95% of patients achieved target maintenance dose of 20 mg/day in 18 weeks while almost 24 weeks were necessary for patients requiring a therapeutic dosage of 40 mg/day [31]. A small minority of patients may need to increase dosage over 40 mg/day to achieve therapeutic response; in these patients dosage can be increased up to 75 mg/day [29, 30].

### Pegvaliase AEs prevention and management

Among all patients participating in pegvaliase clinical studies with induction/titration/maintenance dosing, 9% developed at least one severe hypersensivity reaction, most of which occurred within 1 hour from administration [30]. Considering these data, the presence of a trained caregiver is recommended during the first 6 months of pegvaliase treatment, supervising administration and remaining with the patients for at least 1 hour after injection [29]. Frequency of severe adverse events (AEs) seems to be independent on drug dose or biochemical markers but mainly depends on the patient’s individual immune response, thus it is not possible to predict the patient-related risk. Only patients considered able to understand and follow instruction for managing severe AEs should be enrolled for pegvaliase treatment, or patients with caregivers assuring adequate care [29, 30, 31, 34].

According to tolerability concerns, pegvaliase is only available through a Risk Evaluation and Mitigation Strategies (REMS) program, containing all indications to minimize and manage possible events of hypersensivity. The REMS also includes an educational package for all healthcare professionals, patients, caregivers and observers who are expected to prescribe, use or supervise the administration of pegvaliase, with educational material adapted for all categories, to train them about the recognition and management of severe AEs [29]. According to clinical trials and prescribing information, hypersensivity reactions during induction/titration/maintenance dosing of pegvaliase should be managed by the administration of auto-injectable epinephrine, corticosteroids, antihistamines and/or oxygen. Thus, both patients and caregivers should be trained to administrate epinephrine if necessary and caregivers/observers should also be able to call emergency promptly in case of severe AEs [29, 30].

It is also important to consider that no direct experience on pegvaliase use existed in Italy at the time of consensus, thus statements on adverse events mitigation and management were developed on the basis of published evidence and prescribing information, as all statements on pegvaliase use. Therefore, it is not surprising that, while all other statements on pegvaliase, concerning general guidances for patients’ enrollment and administration, found immediate large consensus, statements on reduction or discontinuation of premedication (#71), and on rechallenge after severe adverse events (#73) elicited a certain diffidence in some experts, since concerning specific decisions during clinical practice. These experts claimed the wish to obtain direct experience before deciding on strategies for management of adverse events. Complying with prescribing information, the SC decided not to rephrase the statements, that were re-discussed in second plenary, particularly pointing out that judgement of the clinical specialist is an essential precondition in decision about premedication and rechallenge. Discussion on this issue finally led to wide acceptance of all statements.

# References

1. Romani C, Manti F, Nardecchia F, Valentini F, Fallarino N, Carducci C, et al. Adult cognitive outcomes in phenylketonuria: explaining causes of variability beyond average Phe levels. Orphanet Journal of Rare Diseas. 2019;14:273.

2. Hagedorn TS, van Berkel P, Hammerschmidt G, Lhotáková M, Saludes RP. Requirements for a minimum standard of care for phenylketonuria: the patients' perspective. Orphanet J Rare Dis. 2013;8:191.

3. van Wegberg AMJ, MacDonald A, Ahring K, Bélanger-Quintana A, Blau N, Bosch AM, et al. The complete European guidelines on phenylketonuria: diagnosis and treatment. Orphanet J Rare Dis. 2017;12:162.

4. Burlina A, Leuzzi V, Spada M, Carbone MT, Paci S, Tummolo A. The management of phenylketonuria in adult patients in Italy: a survey of six specialist metabolic centers. Curr Med Res Opin. 2021;37(3):411-421..

5. Borghi L, Moreschi C, Toscano A, Comber P, Vegni E. The PKU & ME study: A qualitative exploration, through co-creative sessions, of attitudes and experience of the disease among adults with phenylketonuria in Italy. Mol Genet Metab Rep. 2020;23:100585.

6. Albrecht J, Garbade SF, Burgard P. Neuropsychological speed tests and blood phenylalanine levels in patients with phenylketonuria: a meta-analysis. Neurosci Biobehav Rev. 2009;33(3):414–21.

7. Fonnesbeck CJ, McPheeters ML, Krishnaswami S, Lindegren ML, Reimschisel T. Estimating the probability of IQ impairment from blood phenylalanine for phenylketonuria patients: a hierarchical meta-analysis. J Inherit Metab Dis. 2013;36(5):757–66.

8. Prinsen HC, Holwerda-Loof NE, de Sain-van der Velden MG, Visser G, Verhoeven-Duif NM. Reliable analysis of phenylalanine and tyrosine in a minimal volume of blood. Clin Biochem. 2013;46(13–14):1272–5.

9. Prick BW, Hop WC, Duvekot JJ. Maternal phenylketonuria and hyperphenylalaninemia in pregnancy: pregnancy complications and neonatal sequelae in untreated and treated pregnancies. Am J Clin Nutr. 2012;95(2):374-82.

10. Koch R, Hanley W, Levy H, Matalon K, Matalon R, Rouse B, et al. Pediatrics. 2003;112(6 Pt 2):1523-9.

11. Blau N, Hennermann JB, Langenbeck U, Lichter-Konecki U. Diagnosis, classification, and genetics of phenylketonuria and tetrahydrobiopterin (BH4) deficiencies. Mol Genet Metab. 2011;104:S2-9.

12. Romani C, Palermo L, MacDonald A, Limback E, Hall SK, Geberhiwot T. The impact of phenylalanine levels on cognitive outcomes in adults with phenylketonuria: Effects across tasks and developmental stages. Neuropsychology. 2017;31(3):242-54.

13. Anderson PJ, Leuzzi V. White matter pathology in phenylketonuria. Mol Genet Metab. 2010;99 Suppl 1:S3–9.

14. Leuzzi V, Tosetti M, Montanaro D, Carducci C, Artiola C, Carducci C, et al. The pathogenesis of the white matter abnormalities in phenylketonuria. A multimodal 3.0 tesla MRI and magnetic resonance spectroscopy (1H MRS) study. J Inherit Metab Dis. 2007;30(2):209–16.

15. Hood A, Antenor-Dorsey JA, Rutlin J, Hershey T, Shimony JS, McKinstry RC, et al. Prolonged exposure to high and variable phenylalanine levels over the lifetime predicts brain white matter integrity in children with phenylketonuria. Mol Genet Metab. 2015;114(1):19-24.

16. Mastrangelo M, Chiarotti F, Berillo L, Caputi C, Carducci C, Di Biasi C, et al. The outcome of white matter abnormalities in early treated phenylketonuric patients: A retrospective longitudinal long-term study. Mol Genet Metab. 2015;116(3):171-7.

17. Nardecchia F, Manti F, Chiarotti F, Carducci C, Carducci C, Leuzzi V. Neurocognitive and neuroimaging outcome of early treated young adult PKU patients: A longitudinal study. Mol Genet Metab. 2015;115(2-3):84-90.

18. Cleary MA, Walter JH, Wraith JE, Jenkins JP, Alani SM, Tyler K, Whittle D. Magnetic resonance imaging of the brain in phenylketonuria. Lancet. 1994;344(8915):87-90.

19. White DA, Antenor-Dorsey JA, Grange DK, Hershey T, Rutlin J, Shimony JS, et al. White matter integrity and executive abilities following treatment with tetrahydrobiopterin (BH4) in individuals with phenylketonuria. Mol Genet Metab. 2013;110(3):213–7.

20. Pilotto A, Blau N, Leks E, Schulte C, Deuschl C, Zipser C, et al. Cerebrospinal fluid biogenic amines depletion and brain atrophy in adult patients with phenylketonuria. J Inherit Metab Dis. 2019;42(3):398-406.

21. Pilotto A, Zipser CM, Leks E, Haas D, Gramer G, Freisinger P, et al. Phenylalanine Effects on Brain Function in Adult Phenylketonuria. Neurology. 2021;96(3):e399-e411.

22. Grange DK, Hillman RE, Burton BK, Yano S, Vockley J, Fong CT, et al. Phenylketonuria Demographics Outcomes and Safety (PKUDOS) registry; Maternal Phenylketonuria Observational Program (PKU MOMS) sub-registry. Sapropterin dihydrochloride use in pregnant women with phenylketonuria: an interim report of the PKU MOMS sub-registry. Mol Genet Metab. 2014;112(1):9-16.

23. Muntau AC, Adams DJ, Bélanger-Quintana A, Bushueva TV, Cerone R, Chien YH, et al. International best practice for the evaluation of responsiveness to sapropterin dihydrochloride in patients with phenylketonuria. Mol Genet Metab. 2019:127(1):1-11.

24. Teissier R, Nowak E, Assoun M, Mention K, Cano A, Fouilhoux A, et al. Maternal phenylketonuria: low phenylalaninemia might increase the risk of intra uterine growth retardation. J Inherit Metab Dis. 2012;35(6):993-9.

25. Blau, N. Sapropterin dihydrochloride for the treatment of hyperphenylalaninemias. Expert Opin Drug Metab Toxicol. 2013;9(9):1207–18.

26. Burlina A, N Blau N. Effect of BH(4) supplementation on phenylalanine tolerance. J Inherit Metab Dis. 2009;32(1):40-5.

27. Douglas TD, Ramakrishnan U, Kable JA, Singh RH. Longitudinal quality of life analysis in a phenylketonuria cohort provided sapropterin dihydrochloride. Health Qual Life Outcomes. 2013;11:218.

28. Longo N, Siriwardena K, Feigenbaum A, Dimmock D, Burton BK, Stockler S, et al. Long-term developmental progression in infants and young children taking sapropterin for phenylketonuria: a two-year analysis of safety and efficacy. Genet Med. 2015;17(5):365-73.

29. Palynziq^®^ (pegvaliase-pqpz) SmPC. BioMarin Pharmaceutical. https://www.ema.europa.eu/en/documents/product-information/palynziq-epar-product-information_en.pdf

30. Longo N, Dimmock D, Levy H, Viau K, Bausell H, Bilder DA, et al. Evidence- and consensus-based recommendations for the use of pegvaliase in adults with phenylketonuria. Genet Med. 2019;21(8):1851-67.

31. Thomas J, Levy H, Amato S, Vockley J, Zori R, Dimmock D, et al. Pegvaliase for the treatment of phenylketonuria: Results of a long-term phase 3 clinical trial program (PRISM). Mol Genet Metab. 2018;124(1):27–38.

32. Hydery T, Azzopardi Coppenrath V. A Comprehensive Review of Pegvaliase, an Enzyme Substitution Therapy for the Treatment of Phenylketonuria. Drug Target Insights. 2019;13:1177392819857089.

33. Hausmann O, Daha M, Longo N, Knol E, Müller I, Northrup H, Brockow K. Pegvaliase: Immunological profile and recommendations for the clinical management of hypersensitivity reactions in patients with phenylketonuria treated with this enzyme substitution therapy. Mol Genet Metab. 2019;128(1-2):84-91.

34. Zori R, Thomas JA, Shur N, Rizzo WB, Decker C, Rosen O, et al. Induction, titration, and maintenance dosing regimen in a phase 2 study of pegvaliase for control of blood phenylalanine in adults with phenylketonuria. Mol Genet Metab. 2018;125(3):217-27.
